# Quantitative cotyloid fossa thickness and proximity to obturator neurovascular bundle: implications for arthroscopic ligamentum teres reconstruction

**DOI:** 10.1093/jhps/hnad020

**Published:** 2023-08-28

**Authors:** Jacek Mazek, Nader Helmy, Antonio Porthos Salas, Pawel Skowronek, Arkadiusz Madej, John M O´Donnell, Dimitris Dimitriou

**Affiliations:** Clinic of Orthopaedic and Traumatology, Regional Hospital and Kochanowski Medical University, Grunwaldzka 45, Kielce 25-736, Poland; Department of Orthopaedics and Traumatology, Bürgerspital Solothurn, Schöngrünstrasse 42, Solothurn 4500, Switzerland; Hip Arthroscopy of Mexico, Hospital Angeles Valle Oriente, Av. Cto. Frida Kahlo 180, Valle Oriente, San Pedro Garza García, N.L. 66260, Mexico; Clinic of Orthopaedic and Traumatology, Regional Hospital and Kochanowski Medical University, Grunwaldzka 45, Kielce 25-736, Poland; Clinic of Orthopaedic and Traumatology, Regional Hospital and Kochanowski Medical University, Grunwaldzka 45, Kielce 25-736, Poland; Hip Arthroscopy Australia, 21 Erin St, Richmond, Melbourne, VIC, Australia; Department of Orthopedics, University Hospital Balgrist, University of Zurich, Forchstrasse 340, Zürich 8008, Switzerland

## Abstract

The aim of the present study was to report the *in vivo* thickness of the cotyloid fossa at the acetabular ligamentum teres (LT) attachment and investigate the clearance of the obturator neurovascular bundle. Fifty-five consecutive patients undergoing a total hip arthroplasty for hip osteoarthritis were included. The thickness of the cotyloid fossa was measured at the acetabular LT attachment using a standard depth gauge. The minimal distance (clearance) of the obturator neurovascular bundle to the center of the acetabular LT attachment was measured in 7 patients (14 hips) who also underwent a computed tomography angiography. The average thickness of the cotyloid fossa at the acetabular LT attachment was 4.1 ± 2.3 (range: 1–10) mm. The obturator vein was closest to the acetabular LT attachment, but the clearance was more than the defined safe zone of 15 mm in all cases. Based on the current findings, it can be assumed that bone anchors might not be suitable for fixation of the graft in LT reconstruction (LTR) and an alternative implant such as a cortical button should be considered. Acetabular fixation of the graft with a 12-mm cortical button is relatively safe concerning injury to obturator neurovascular structures. The results of the present study provide a better understanding of the cotyloid fossa anatomy and might be relevant for surgeons who perform arthroscopic LTR.

## INTRODUCTION

Before the advent and widespread application of hip arthroscopy, limited data were available regarding pathologies and treatment of ligamentum teres (LT) injuries, originally believed to be a vestigial structure. Recently, LT is gaining increased attention as several anatomic [1] and biomechanical studies [2] revealed its important role as static restraint of the hip joint, providing end-range stabilization of hip rotation, predominantly at 90° or greater of hip flexion [3]. LT is an intra-articular ligament that arises from the transverse acetabular ligament and inferior aspect of the cotyloid fossa and inserts into the fovea femoris capitis [1]. In patients with osseous risk factors for hip instability such as acetabular dysplasia, the LT functions as an important hip joint restraint, not only primarily in hip flexion/external rotation but also in extension/internal rotation [2, 3].

Evidence suggests that LT tears might cause groin and thigh pain and have been reported in up to 51% of patients undergoing a hip arthroscopy [4], with even higher rates identified in patients with osseous deficiencies or ligamentous laxity contributing to hip microinstability [5, 6]. Treatment of LT injuries has been a subject of recent debate. In most of the patients, debridement or radiofrequency ablation of both partial and complete LT tears demonstrates satisfactory results [7]. However, in a relatively small subset of patients, who have persistent pain and instability following arthroscopic debridement of LT, an LT reconstruction (LTR) might be indicated to restore stability and increase function [8]. The LTR was first described by Simpson *et al*. [9] in 2011 as graft implantation that spans between the femoral head and cotyloid fossa through two tunnels, thereby restoring the function of the native LT. As an increasing body of evidence reports favorable outcomes following LTR [10], its indication has been expanded not only to patients with failed hip arthroscopy but also to patients with hip pain and instability (Beighton score >4 points) in the presence of LT tear >50% and normal acetabular coverage [11].

Despite the increasing popularity and the promising outcomes of LTR, it is a technically demanding procedure and given the thin cortical bone of the cotyloid fossa and its close proximity to the arthroscopically not visible obturator neurovascular bundle, caution is required when drilling the acetabular tunnel to avoid damage to these structures. Previous anatomical studies of the pelvis focused on determining the structures in danger during acetabular screw placement during hip replacement [12] and labral repair [13]. To date and to the best knowledge of the authors, only one cadaveric study [14] with nine fresh-frozen human pelvises described a safe acetabular tunnel–drilling technique in LTR, particularly concerning avoiding damage to the obturator neurovascular bundle, especially in the case of violation of the medial cotyloid wall. However, the *in vivo* relationship between the cotyloid fossa and the pelvis neurovascular structures has never been investigated. Therefore, the aim of the present study was to report the thickness of the cotyloid fossa at the acetabular LT attachment in patients who underwent a total hip arthroplasty (THA) through the direct anterior approach (DAA) and investigate the proximity of the obturator neurovascular bundle to the acetabular LT attachment using a computed tomography (CT) angiography. The study hypothesis was that the obturator neurovascular structure would have a safe minimum distance of at least 15 mm from the acetabular LT footprint in the case of medial wall violation.

## MATERIALS AND METHODS

### Study design and patient selection

The present single-center, prospective study was approved by the institutional internal review boards and ethical committees. Following written informed consent, consecutive patients planned to undergo a THA for an end-grade (Tonnis Grade 3) hip osteoarthritis were included.

### Measurement of the cotyloid wall thickness

**Fig. 1.**
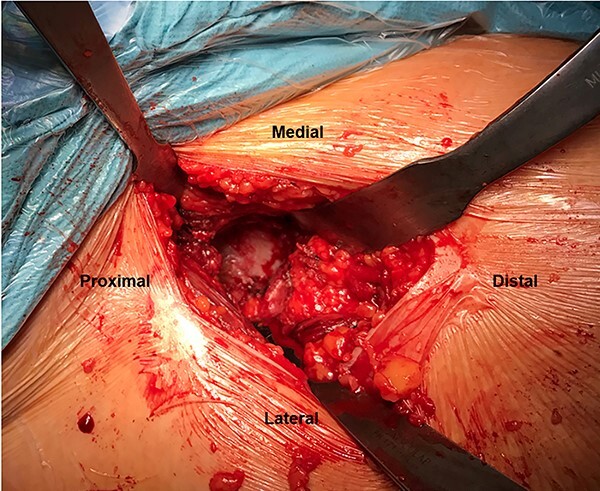
An intraoperative photograph of a right hip. A DAA to the hip joint was performed, and three retractors were placed to achieve adequate visualization of the acetabulum.

**Fig. 2.**
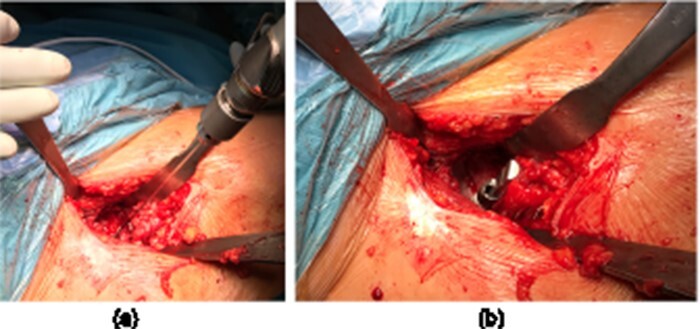
An intraoperative photograph of a right hip demonstrating (**a**) the direction of the 2-mm Kirschner wire insertion and (**b**) the measurement of the cotyloid fossa thickness at the acetabular LT attachment using a standard depth gauge.

All the patients underwent a THA through the DAA, which provides characteristically excellent access and visibility to the acetabulum [15]. The surgery was performed without a traction table, and an incision was made over the hip joint, 2 cm distal and lateral to the anterosuperior iliac spine. The internervous plane between the tensor fasciae lata/sartorius superficially and gluteus medius/rectus femoris deeply was used. Once the hip joint has been fully exposed, the arthritic femoral head was removed and three retractors were used to achieve adequate visualization of the acetabulum. The first sharp retractor was placed on the posterolateral acetabulum, the second sharp was placed over the anterior column and a blunt retractor was placed to expose the medial portion of the acetabulum. These three retractors were placed at 90° angles to each other (Fig. 1). The soft tissues were removed from the cotyloid fossa, including pulvinar and LT, with extra attention paid to identifying the acetabular LT attachment. A 2-mm Kirschner wire was passed through the center of the acetabular LT attachment until the medial acetabular was perforated. The direction of the Kirschner wire insertion was similar to the one used during LTR and was performed by a senior hip surgeon (J.M.) who performs more than 100 THA and 150 hip arthroscopies (including LTR) yearly (Fig. 2a). The thickness of the cotyloid fossa was then measured at the acetabular LT attachment using a standard depth gauge (Fig. 2b). Drilling of the acetabulum in sclerotic areas is a common practice for better integration of the implant.

### Measurement of the minimal distance of LT acetabular insertion to obturator neurovascular bundle

**Fig. 3.**
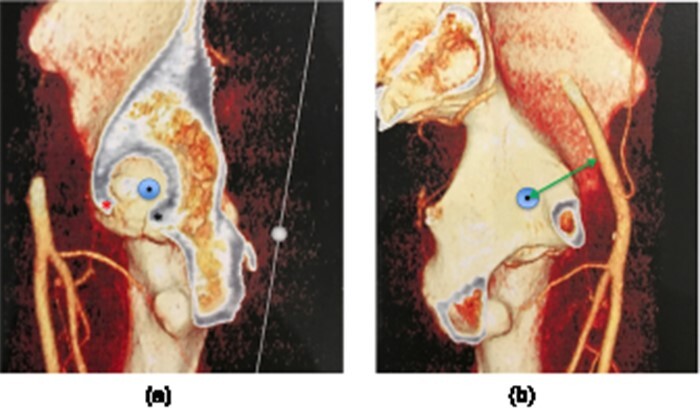
Three-dimensional reconstruction of a CT angiography demonstrating the LT acetabular attachment (large circle). (A) View of the cotyloid fossa and (B) view of the quadrilateral plate of the pelvis demonstrating the clearance distance (arrow) between the center of the acetabular LT tunnel (small circle) and obturator vein.

In order to determine the anatomical relationship between the LT acetabular insertion and the obturator neurovascular bundle, a CT angiography was performed in seven patients (14 hips). The LT acetabular insertion and its extension on the quadrilateral plate of the pelvis were identified by a senior hip surgeon (J.M.). The obturator artery and vein were also identified, and the minimal distance between the center of the acetabular LT attachment and the obturator artery and vein, defined as clearance, was measured. The measurements were performed using OsiriX DICOM viewer software version 5.7 (OsiriX Foundation, Geneva, Switzerland) using the CT bone view using the three-dimensional multiplanar reconstruction tool (Fig. 3a and b).

Since the graft (usually autologous semitendinosus of 6 mm diameter) is typically looped over a 12-mm cortical button fixation device, which is passed through the acetabular tunnel and then flipped against the medial wall of the pelvis, the safe distance of the acetabular graft tunnel to the obturator neurovascular bundle was defined at 15 mm for the present study. This is the preferred technique performed by the senior author.

### Statistical analysis

Descriptive statistics used mean, standard deviation, range and percentages to present the data. All parameters were tested using the Kolmogorov–Smirnov test for normality. A two-tailed unpaired *t*-test was used to compare the thickness of the cotyloid fossa between genders. A Pearson correlation was applied to detect potential relationships between the thickness of the cotyloid fossa and age, height, weight or body mass index (BMI). The level of significance was set at *P* = 0.05. Statistical analysis was performed using SPSS version 23 software (SPSS Inc., Chicago, IL, USA).

## RESULTS

### Patient demographics

A total of 55 consecutive patients (55 hips, left: 27) undergoing a THA for an end-grade (Tonnis Grade 3) hip osteoarthritis (male: 29) with an average age of 59 ± 14 (range: 31–86) years and a BMI of 27.1 ± 4.6 (range: 18–43) kg/m^2^ were included. Patients with severe osteophytes or radiographic obliteration of the cotyloid fossa were excluded. Seven patients (13%) agreed to also receive a CT angiography preoperatively, whereas the rest of the patients did not want to undergo a CT scan.

### Measurement of the cotyloid wall thickness and minimal distance of LT acetabular insertion to obturator neurovascular bundle

**Fig. 4.**
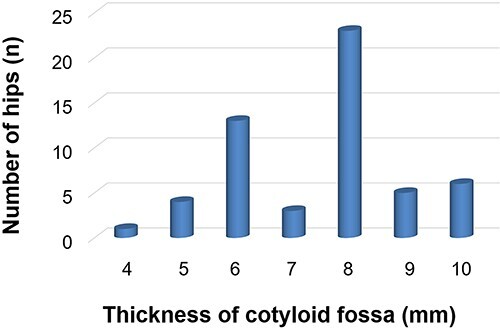
A histogram summarizing the distribution of the cotyloid fossa thickness at the acetabular LT attachment.

The average thickness of the cotyloid fossa at the center of the acetabular LT attachment was 7.5 ± 1.5 (range: 4–10) mm. In 5/55 (9%) of the patients, the cotyloid fossa thickness was ≤5 mm (Fig. 4). No significant difference regarding the thickness of the cotyloid fossa was observed between men (7.2 ± 1.7 mm) and women (7.5 ± 1.0 mm) (*P* =0.45). The obturator vein was closest to the acetabular LT attachment, but the clearance was more than the defined safe zone of 15 mm in all patients. No significant correlations were found between the thickness of the medial wall and age (*r* = −0.1, *P* = 0.5), weight, (*r* = 0.2, *P* = 0.1), height (*r* = 0.1, *P* = 0.3) or BMI (*r* = 0.2, *P* = 0.14).

## DISCUSSION

During the past decade, there has been a growing interest in the functional role of LT in hip stability [3]. An increasing body of evidence suggests that symptomatic LT injuries could be treated with arthroscopic debridement or radiofrequency ablation with satisfactory results [7]. However, in a subgroup of patients, which experience persistent groin pain or instability, an LTR can be performed. Despite the increasing availability and the promising outcomes of LTR, it is a technically demanding procedure and caution should be exercised, especially when drilling the acetabular tunnel or flipping the cortical button against the medial acetabular wall, due to its close proximity to the obturator neurovascular bundle. Although a few cadaveric studies investigated the structures at danger during acetabular screw placement during THA [12], labral repair [7] and, recently, LTR [14], there is a paucity of data regarding the *in vivo* relationship between the obturator neurovascular bundle and acetabular LT attachment, especially in the case of perforation of the medial cotyloid wall. Therefore, the aim of the present study was to investigate the thickness of the cotyloid fossa at the acetabular LT attachment and investigate clearance of the obturator neurovascular bundle. The most important finding of the present study was that the cotyloid wall thickness varies from 4 to 10 mm, independent of gender, height, age or BMI, and the obturator vein (the structure with the closest proximity to the acetabular LT insertion) has a clearance of at least 15 mm in all patients.

Over the past years, LT is gaining increased attention as several anatomical [1] and biomechanical studies [2] revealed its important role as static restraint of the hip joint. However, limited data are available regarding the *in vivo* thickness of the cotyloid fossa, which recently became relevant for stable graft fixation in LTR. In a laboratory study of 10 human cadavers, Mikula *et al*. [1] identified six distinct LT attachments on the acetabulum (transverse, anterior and posterior margins of the acetabular notch and three cotyloid fossa attachments: ilium, ischium and pubis) with different thickness ranging from 6 mm on average at the ischial attachment to 11.3 mm at the anterior attachment. The present study measured the *in vivo* thickness of the cotyloid fossa at the center of the acetabular LT attachments in 55 patients who underwent a THA for hip osteoarthritis and reported an average thickness of 4.1 ± 2.3 mm, which was not correlated with gender, age, height or BMI. In some patients, the cotyloid fossa was very thin (1 mm in 9% and 2 mm in 26%). Since the most available hip joint suture anchors are more than 5 mm in length [16], a medial wall perforation, with possible insufficient fixation, is to be expected in about 63% of the patients, suggesting that bone anchors might not be suitable for fixation of the graft in LTR, and an alternative implant such as cortical button should be considered. Nevertheless, in some patients, the cortical bone at the cotyloid fossa is very thin, and therefore, an excessive tightening of the graft should not be performed to avoid cortical button cut through the bone, as the maximal load to failure of the cortical button is positively correlated with the tunnel diameter and cortical thickness [17].

Several studies reported that iatrogenic injury to vital pelvic structures might occur during acetabular drilling for screws placement in THA [12] or anchor placement during labral repair [7]. Based on the safe zone system in the study by Waliewski *et al.* [12] for acetabular drilling, the posterior (both inferior and superior) quadrants are considered safe, whereas the anterior quadrants should be avoided due to their close proximity to major neurovascular structures. However, most of the cotyloid fossa is located in the anteroinferior quadrant, and violation of the medial cotyloid wall presents a risk of obturator neurovascular bundle injury. In a cadaveric study, Brady *et al*. [14] simulated a transfemoral acetabular tunnel drilling for LTR (diameter: 2.9 mm) in nine human cadavers in different femur positions and reported that the obturator bundle was avoided in 100% of the simulations, with a mean clearance from the obturator bundle of 10.0 ± 4.9 mm and an average distance of the acetabular tunnel to the native LT attachment of 6.6 ± 3.2 mm. The present study using a CT angiography demonstrated that the average clearance of the native LT acetabular attachment center to the neurovascular bundle was more than 15 mm in all cases, suggesting that acetabular fixation of the graft during LTR with a 12-mm cortical button fixation is a relatively safe procedure concerning injury to the obturator neurovascular bundle.

The present study should be interpreted in light of its potential limitations, mostly inherent to the limited number of patients (7 patients, 14 hips) who agreed to perform a CT angiography. Further studies with a larger subject size might be necessary to confirm the findings of the present study. Despite the relatively small sample size, it is still comparable with the sample size of several cadaveric studies [12–14], and it is the only available *in vivo* study investigating the close relationship of the obturator neurovascular bundle to the acetabular LT attachment. Additionally, all the subjects in the present study were Caucasians. Therefore, the results of the present study might not reflect the cotyloid fossa anatomy of the other ethnicities. Finally, the anatomical measurement was performed in patients with severe hip osteoarthritis and therefore might not reflect the anatomy of the patients with hip instability. Although patients with osteophytes or obliteration of the cotyloid fossa were excluded, the osseous acetabular morphology might have been disturbed due to hip osteoarthritis.

In conclusion, the current study is the only available study in the literature to report the thickness of the cotyloid fossa at the acetabular LT attachment and investigate the *in vivo* clearance of the obturator neurovascular bundle. Based on the current findings, it can be assumed that bone anchors might not be suitable for graft fixation in LTR and an alternative implant such as a cortical button should be considered. Acetabular fixation of the graft during LTR with a 12-mm cortical button is relatively safe concerning injury to the obturator neurovascular structures. The results of the present study provide a better understanding of the cotyloid fossa anatomy and might be relevant for surgeons who perform arthroscopic LTR.

## Data Availability

The authors confirm that the data supporting the findings of this study are available within the article.

## LEVEL OF EVIDENCE

Prospective cohort study, Level III.

## References

[1] Mikula JD, Slette EL, Chahla J et al. Quantitative anatomic analysis of the native ligamentum teres. *Orthop J Sports Med* 2017; 5.doi: 10.1177/2325967117691480 PMC5347434 28321426

[2] Rosinsky PJ, Shapira J, Lall AC et al. All about the ligamentum teres: from biomechanical role to surgical reconstruction. *J Am Acad Orthop Surg* 2020; 28: e328–39. 31860583 10.5435/JAAOS-D-19-00352

[3] Martin HD, Hatem MA, Kivlan BR et al. Function of the ligamentum teres in limiting hip rotation: a cadaveric study. *Arthroscopy* 2014; 30: 1085–91. 24908256 10.1016/j.arthro.2014.04.087

[4] Botser IB, Martin DE, Stout CE et al. Tears of the ligamentum teres: prevalence in hip arthroscopy using 2 classification systems. *Am J Sports Med* 2011; 39: 117S–25S. 21709041 10.1177/0363546511413865

[5] Domb BG, Lareau JM, Baydoun H et al. Is intraarticular pathology common in patients with hip dysplasia undergoing periacetabular osteotomy? *Clin Orthop Relat Res* 2014; 472: 674–80. 24096455 10.1007/s11999-013-3140-2 PMC3890175

[6] Domb BG, Martin DE, Botser IB. Risk factors for ligamentum teres tears. *Arthroscopy* 2013; 29: 64–73. 23276414 10.1016/j.arthro.2012.07.009

[7] Amenabar T, O’Donnell J. Successful treatment of isolated, partial thickness ligamentum teres (LT) tears with debridement and capsulorrhaphy. *Hip Int* 2013; 23: 576–82. 23934903 10.5301/hipint.5000072

[8] Menge TJ, Mitchell JJ, Briggs KK et al. Anatomic arthroscopic ligamentum teres reconstruction for hip instability. *Arthrosc Tech* 2016; 5: e737–42. 27709030 10.1016/j.eats.2016.02.036 PMC5039780

[9] Simpson JM, Field RE, Villar RN. Arthroscopic reconstruction of the ligamentum teres. *Arthroscopy* 2011; 27: 436–41. 21292435 10.1016/j.arthro.2010.09.016

[10] Knapik DM, Farivar D, Kunze KN et al. Indications and outcomes after ligamentum teres reconstruction: a systematic review. *Arthrosc Sports Med Rehabil* 2021; 3: e939–49. 34195664 10.1016/j.asmr.2021.01.023 PMC8220633

[11] Rosinsky PJ, Annin S, Maldonado DR et al. Arthroscopic ligamentum teres reconstruction: minimum 2-year patient-reported outcomes with subanalysis of patients with Ehlers-Danlos syndrome. *Arthroscopy* 2020; 36: 2170–82. 32360268 10.1016/j.arthro.2020.04.028

[12] Wasielewski RC, Cooperstein LA, Kruger MP et al. Acetabular anatomy and the transacetabular fixation of screws in total hip arthroplasty. *J Bone Joint Surg Am* 1990; 72: 501–8. 2324135

[13] Gereli A, Kocaoglu B, Ulku KT et al. Are pelvic anatomical structures in danger during arthroscopic acetabular labral repair? Definition of safe bone depth. *Knee Surg Sports Traumatol Arthrosc* 2017; 25: 45–9. 26419377 10.1007/s00167-015-3797-z

[14] Brady AW, Chahla J, Locks R et al. Arthroscopic reconstruction of the ligamentum teres: a guide to safe tunnel placement. *Arthroscopy* 2018; 34:144–51. 29203379 10.1016/j.arthro.2017.08.308

[15] Holst DC, Yang CC. Surgical anatomy of the direct anterior approach for total hip arthroplasty. *Ann Jt* 2018; 3: 23.

[16] Douglass NP, Behn AW, Safran MR. Cyclic and load to failure properties of all-suture anchors in synthetic acetabular and glenoid cancellous bone. *Arthroscopy* 2017; 33: 977–85.e5. 28132809 10.1016/j.arthro.2016.11.022

[17] Massey PA, Caldwell C, Vauclin CP et al. The ideal cortical button location on the lateral femur for anterior cruciate ligament suspensory fixation is 30 mm proximal to the lateral epicondyle. *Arthrosc Sports Med Rehabil* 2021; 3: e1255–62. 34712961 10.1016/j.asmr.2021.03.018 PMC8527268

